# The Akt signaling pathway is required for tissue maintenance and regeneration in planarians

**DOI:** 10.1186/s12861-016-0107-z

**Published:** 2016-04-11

**Authors:** T. Harshani Peiris, Daniel Ramirez, Paul G. Barghouth, Néstor J. Oviedo

**Affiliations:** Department of Molecular and Cell Biology, School of Natural Sciences, University of California, 5200 North Lake Road, Merced, CA 95343 USA; Quantitative and Systems Biology Graduate Program, University of California, Merced, CA 95343 USA; Health Sciences Research Institute, University of California, Merced, CA 95343 USA

**Keywords:** Planarians, Regeneration, Akt, Stem cells

## Abstract

**Background:**

Akt (PKB) is a serine threonine protein kinase downstream of the phosphoinositide 3-kinase (PI3K) pathway. In mammals, Akt is ubiquitously expressed and is associated with regulation of cellular proliferation, metabolism, cell growth and cell death. Akt has been widely studied for its central role in physiology and disease, in particular cancer where it has become an attractive pharmacological target. However, the mechanisms by which Akt signaling regulates stem cell behavior in the complexity of the whole body are poorly understood. Planarians are flatworms with large populations of stem cells capable of dividing to support adult tissue renewal and regeneration. The planarian ortholog *Smed-Akt* is molecularly conserved providing unique opportunities to analyze the function of Akt during cellular turnover and repair of adult tissues.

**Results:**

Our findings abrogating *Smed-Akt* with RNA-interference in the planarian *Schmidtea mediterranea* led to a gradual decrease in stem cell (neoblasts) numbers. The reduced neoblast numbers largely affected the maintenance of adult tissues including the nervous and excretory systems and ciliated structures in the ventral epithelia, which impaired planarian locomotion. Downregulation of *Smed-Akt* function also resulted in an increase of cell death throughout the animal. However, in response to amputation, levels of cell death were decreased and failed to localize near the injury site. Interestingly, the neoblast mitotic response was increased around the amputation area but the regenerative blastema failed to form.

**Conclusions:**

We demonstrate Akt signaling is essential for organismal physiology and in late stages of the Akt phenotype the reduction in neoblast numbers may impair regeneration in planarians. Functional disruption of *Smed-Akt* alters the balance between cell proliferation and cell death leading to systemic impairment of adult tissue renewal. Our results also reveal novel roles for Akt signaling during regeneration, specifically for the timely localization of cell death near the injury site. Thus, Akt signaling regulates neoblast biology and mediates in the distribution of injury-mediated cell death during tissue repair in planarians.

**Electronic supplementary material:**

The online version of this article (doi:10.1186/s12861-016-0107-z) contains supplementary material, which is available to authorized users.

## Background

The protein kinase Akt also known as PKB, regulates multiple cellular functions including proliferation, survival, and growth during embryonic development and adult tissue homeostasis [1–5]. In mammals, Akt expression is widely distributed across the body and includes three isoforms, Akt-1-3 (PKBα, ß, and δ, respectively) [1–3]. Akt is evolutionary conserved in both its molecular structure and function among vertebrate and invertebrate organisms [4–7]. Across metazoans, Akt signaling integrates local and systemic information central to cellular and organismal physiology.

Akt regulates adult stem cell proliferation, migration and apoptosis and its deregulation has been implicated in the progression of cancer, diabetes, and aging [1–4, 8–10]. Conditional deletions and transgenic approaches have revealed key aspects of Akt signaling in hematopoietic, epithelial, neural and other tissues [2, 3, 11–14]. Nonetheless, there is limited understanding of how Akt signaling controls the response of stem cells during cellular turnover and tissue injury in the complexity of the whole organism. This paucity is likely due to the ubiquitous nature of this signaling pathway and the difficulty of analyzing stem cells in their natural environment during physiological cell turnover and regeneration in conventional animal models [15–17].

Thus, we sought to investigate Akt function during cellular turnover and injury using the planarian flatworm *Schmidtea mediterranea*. This organism is well known for its stem cell-based regenerative capability. Planarians contain an abundant and accessible population of somatic adult stem cells called neoblasts [18–21]. The neoblasts are the only dividing cells in planarians and constantly proliferate to repair tissues and support systemic cellular turnover [21]. Recently, we described that the genome of *S. mediterranea* contains a single Akt ortholog termed *Smed-Akt*, which affects cell division and impairs planarian locomotion [22]. This study defined the role of *Smed-Akt* in abnormal cell proliferation triggered by the abrogation of the phosphatase PTEN, an upstream component of the Akt signaling pathway, which is highly mutated in human cancers.

Here we report on an extended RNA-interference (RNAi) strategy that disrupts *Smed-Akt* in the whole organism, to analyze its function on the response of neoblasts during systemic cell turnover and tissue repair. Our results show, *Smed-Akt* abrogation leads to a gradual decline in the number of neoblasts, accompanied by massive cell death that affects cellular turnover and maintenance of adult tissues. We also found that impaired locomotion in the *Smed-Akt* phenotype is due to the disruption of cilia maintenance in the ventral epithelium. Intriguingly, large-scale tissue injury is capable of reducing the high levels of *Smed-Akt(RNAi)*-induced levels of cell death, while increasing neoblast proliferation near the wound site however, animals failed to complete the formation of the regenerative blastema. Thus, our results reveal novel roles for Akt signaling during systemic cell turnover and large-scale regeneration of adult tissues.

## Results

### Smed-Akt is Required for Proper Neoblast Function

**Fig. 1:**
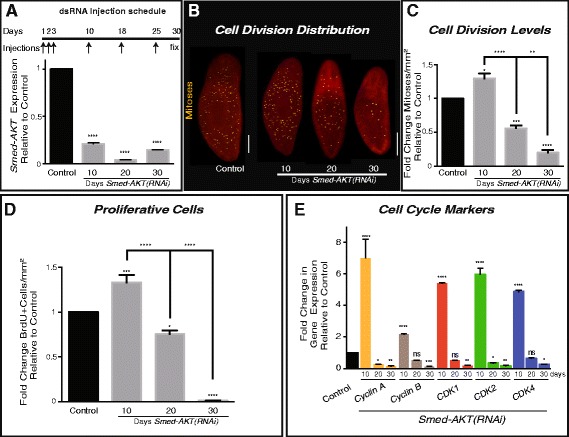
Downregulation of *Smed-Akt(RNAi)* reduces neoblast proliferation. **a** The dsRNA microinjection schedule is depicted on top. A total of 6 injections (arrows) were performed during a 30 day (black line) period. The RNAi efficiency of the dsRNA microinjections was tested with quantitative PCR(qPCR) on days 10, 20 and 30 after the first dsRNA injection, showing significant down-regulation relative to control. **b** Whole-mount immunostaining of control and *Smed-AKT(RNAi)* worms, using anti-phosphorylated histone H3 (H3P) antibody to label the distribution of cell division at days 10, 20 and 30 after first dsRNA injection. Yellow dots represent mitotic cells. Scale bar 200μm. **c** H3P-signal quantification, represents fold change of cell division (mitoses per mm^2^) relative to the control at days 10, 20 and 30 after *Smed-AKT(RNAi)*. A significant increase in cell division was observed 10 days post first injection, which dramatically diminishes by day 30. **d** Quantification of fold change in bromodeoxyuridine (BrdU) positive cells relative to the control at days 10, 20 and 30 after first injection. **e** Relative levels of gene expression, fold change, of cell cycle markers 10, 20 and 30 days after first *Smed-AKT(RNAi)* injection. All analyses were performed using two or more experimental sets with 10 or more animals per experiment at various time points after first dsRNA injection. For qPCR analysis, gene expressions are relative to the ubiquitously expressed clone *H.55.12e*. Graphs represent mean ± s.e.m. of three of more independent experiments. *P* values *** < 0.005 and **** < 0.0001, were calculated by two way ANOVA

Our previous studies identified in the planarian *Schmidtea mediterranea* genome a single Akt ortholog (*Smed-Akt*) to the mammalian Akt2/PKB-ß [22]. *Smed-Akt* is widely expressed in neoblasts and differentiated cells and functional downregulation with RNA-interference [*Smed-Akt(RNAi)*] led to the reduction of neoblast numbers and loss of planarian locomotion [22]. To test whether Akt signaling plays additional roles in the regulation of cellular turnover and tissue regeneration in the adult body, we designed an RNAi protocol consisting of six dsRNA microinjections that effectively downregulated (8.4 folds) *Smed-Akt* expression over the span of 30 days (Fig. 1a).

Neoblast division was visualized through whole-mount immunostaining against the α–phosphorylated histone-3 (H3P) antibody, which labels cells in G2/M phase of the cell cycle (observed as yellow dots in Fig. 1b). Animals subjected to *Smed-Akt(RNAi)* initially displayed an important increase in neoblast division (~0.75 fold) 10 days post RNAi initiation, which was followed by a gradual decline in mitoses, reaching ~ five-fold decrease by day 30, when compared to control (Fig. 1b, c). Importantly, all samples were processed either before or a few days after injection to avoid the possibility of injury-induced increase in mitotic activity. To further characterize the effects of Akt downregulation on the cell cycle dynamics, we evaluated the incorporation of the bromodeoxyuridine analog (BrdU) every ten days for one month (Fig. 1d). BrdU is incorporated during the S phase of the cell cycle and remains in the cell through multiple rounds of cell division, albeit at lower concentrations in each successive cell generation. Control and *Smed-Akt(RNAi)* animals were exposed to a single BrdU pulse at different time points after the first dsRNA injection (i.e. 10, 20, and 30 days) and after 12 h samples were processed as previously described [23]. Consistent with the mitotic counts, BrdU positive cells increased in the first 10 days after *Smed-Akt(RNAi)* and gradually decrease to almost undetectable levels after one month of RNAi treatment (Fig. 1d). We also found a consistent trend in the expression of genes associated with cell cycle regulation (i.e. *cyclin A, cyclin B, CDK1, CDK2,* and *CDK4*), which showed general increase during the first 10 days and dramatically decreases in the successive days, further confirming our observations in mitotic activity and BrdU labeling upon *Smed-Akt(RNAi)* (Fig. 1e). The early increase in gene expression and proliferative cells upon *Smed-Akt* downregulation implies that the phenotype most likely starts before day 10. Our results suggest that *Smed-Akt* is essential to maintain the appropriate number of proliferating neoblast during tissue renewal in adult planarians.

**Fig. 2:**
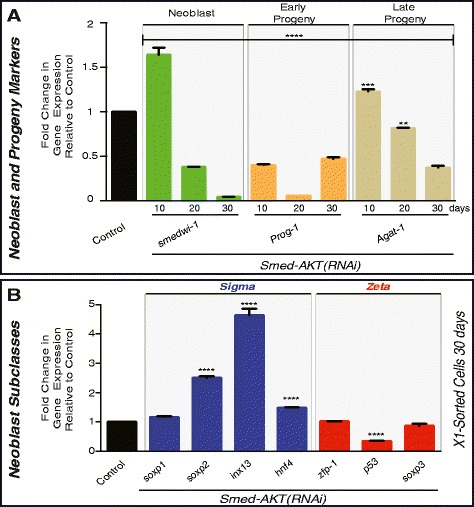
Akt regulates expression of neoblast and progeny markers. **a** Gene expression levels for markers of neoblasts (*smedwi-1)* and neoblast early (*Prog-*1) and late (*AGAT-1*) progeny were evaluated with qPCR at days 10, 20 and 30 after first dsRNA injection and represented in fold change relative to each sample's individual control. **b** Fold change in gene expression levels involving markers of neoblast subpopulations sigma (blue) and zeta (red). The mRNA levels were measured in X1 sorted cells at day 30 after first injection was used to determine gene expression of *Smed-AKT(RNAi)* animals. All values are relative to equal number (~20,000) of X1 cells sorted from control-water injected animals at 30 days. Gene expressions are all relative to the internal control, the ubiquitously expressed clone *H.55.12e*. Graphs represent mean ± s.e.m. of triplicated samples of two or more biological replicates with at least ten animals per experiment. *P* values ** < 0.001, *** < 0.0005 and **** < 0.0001, were calculated by two way ANOVA

To assess whether the effects of *Smed-Akt(RNAi)* are restricted to cell cycle events, we analyzed the expression of markers associated with neoblasts and the early and late division progeny (e.g. *smedwi-1*, *Prog-1, and Agat-1*, respectively). This analysis revealed a dramatic decrease in the expression of markers associated with neoblasts and their progeny (Fig. 2a). Interestingly, the pattern of expression for *smedwi-*1 and *Agat-*1 were similar to that previous observed in cell cycle genes, while the marker for the early neoblast progeny (*prog-1*) followed a somewhat different pattern, characterized by a strong downregulation from the beginning of *Smed-Akt(RNAi)*. Whole mount fluorescent in situ hybridization (FISH) with markers of neoblasts and the post-mitotic progeny one month after *Smed-Akt(RNAi)* further confirmed the quantitative PCR (qPCR) results and showed a generalized reduction in gene expression throughout the body, after *Smed-Akt(RNAi)* (Additional file 1).

Proliferative neoblasts are contained within the irradiation sensitive X1 population and can be isolated via flow cytometry cell sorting (FACS) [24]. Recently, cells within the X1 population were classified into two functionally distinct subclasses, the zeta- and sigma-neoblasts [25]. FACS was used to isolate equal number of X1 cells from both control and *Smed-Akt(RNAi)* animals after one month of RNAi. We used a larger number of experimental animals to obtain comparable amount of FACS-isolated cells between both groups. The total RNA extracted was processed to evaluate levels of expression of markers of the sigma and zeta populations (Fig. 2b). Markers of the sigma neoblast subpopulations tend to increase upon *Smed-Akt(RNAi),* while there little to no change in the zeta subclass, suggesting the effect of *Smed-Akt(RNAi)* is not homogeneously distributed among the neoblasts. Thus, additional experiments are required to further define the differences between gene expression in the neoblast subclasses and to understand these implications. Altogether, our results indicate that *Smed-Akt* is essential for the appropriate expression of neoblast and progeny markers and suggests that a gradual depletion in the number of neoblasts takes place after *Smed-Akt(RNAi)*.

### Smed-Akt is a Critical Regulator of Cell Death in Planarians

**Fig. 3:**
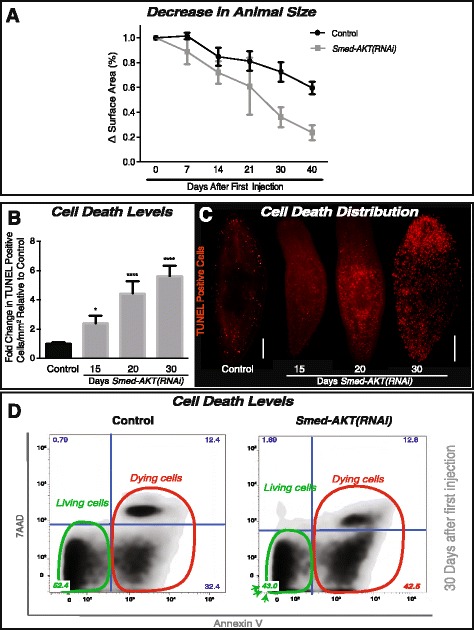
The impairment of Akt leads to increased cell death in *S. mediterranea*. **a** The reduction in animal size over time was recorded as the difference in surface area (whole animal) expressed in percentage. Surface area measurements were followed for over 40 days of starvation in both the control and *Smed-AKT (RNAi)* animal groups. Graph represent the mean ± s.e.m of five independent time courses with ten or more animals per experiment. **b** Quantification of TUNEL-positive nuclei reveals a gradual increase in cell death from day 15 (~2.5 fold) to 30 days (~6 fold) post first injection relative to its individual control. Graph represent mean ± s.e.m of three biological replicates consisting of five or more animals per experiment. *P*-value * <0.05 and **** <0.0001, by one way-ANOVA. **c** Whole-mount immunostaining labeling TUNEL-positive nuclei (cell death) with control on the left and *Smed-AKT(RNAi)* days 15, 20 and 30 on the right. Each time point consisted of at least two biological replicates with 5 or more animals each. Scale bar 200μm. **d** Flow cytometry analysis of Annexin V and 7 AAD expression, reveals the frequency distribution between living and dead cells. Annexin V-7 AAD quadrant indicates live cells (green circle). Annexin V + 7 AAD and Annexin V + 7 AAD+ indicates cells that are in early and late stages of cell death, respectively (red circle). The numbers in each quadrant indicate the percentage of cells with that staining profile. All experiments were independently repeated three times with at least ten animals each time

A fine balance between stem cell proliferation and programmed cell death enables physiological cellular turnover that supports maintenance and growth of adult tissues [26]. Over a 40 days starvation period, animals subjected to *Smed-Akt(RNAi)* exhibited a ~3 fold reduction in surface area compared to the control group (0.23 ± 0.08 vs 0.72 ± 0.07 mm^2^, respectively) (Fig. 3a). These results together with the reduction in neoblast proliferation suggests that the *Smed-Akt(RNAi)* phenotype may be accompanied by increased levels of cell death, contributing to an accelerated reduction in animal size over time, and death by ~45 days after the first injection.

Akt function has been implicated in cellular pro-survival mechanisms [14, 27–29]. Thus, we examined possible roles of *Smed-Akt* as regulator of cell death in planarians by analyzing levels of cell death after RNAi treatment. First, spatial distribution of cell death in the whole body was evaluated using the terminal deoxynucleotidyl transferase dUTP nick end-labeling (TUNEL) assay [30]. These experiments revealed that *Smed-Akt (RNAi)* double the number of TUNEL positive cells, 15 days after the first dsRNA injection and it gradually increase to about threefold by day 30 of the RNAi treatment (Fig. 3b). Despite a slight increase in TUNEL positive cells by day 10 of *Smed-Akt(RNAi)* (data not shown), the predominant increase in cell death was observed around day 15 after the first dsRNA injection. TUNEL positive cells appeared indistinctively scattered along the planarian body at all times, suggesting this is a generalized event, most likely involving both neoblasts and differentiated cells (Fig. 3c). Next, we performed flow cytometry analysis using Annexin V and 7AAD staining [31, 32]. This experiment confirmed the high levels of cell death induced by *Smed-Akt(RNAi)* may involve apoptosis and necrosis (Fig. 3d). The increased levels of cell death in *Smed-Akt (RNAi)* together with the overall animal size reduction indicates that Akt is an important regulator of cellular turnover. The mechanisms are not entirely clear however, it is possible that the impairment in tissue renewal may result from either (1) an initial reduction in neoblast numbers that fail to support homeostasis or (2) generalized cell death events that impact both neoblasts and differentiated cells alike.

### *Smed-Akt* Regulates the Maintenance of Differentiated Tissues

**Fig. 4:**
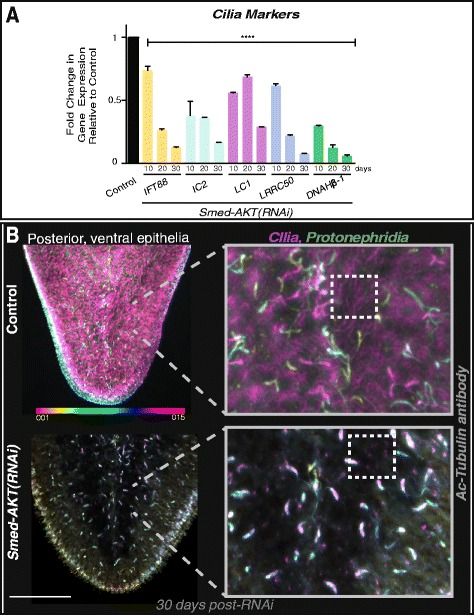
Akt is required for the maintenance of cilia in the ventral epithelia. **a** Gene expression levels of cilia specific markers at days 10, 20 and 30 after *Smed-AKT(RNAi)* relative to the control. Analysis of markers corresponding to intraflagellar transport machinery (IFT88) and flagellar beating (IC2, LC1, DNAHβ-1 and LRRC50) reveal a marked reduction of gene expression as early as day 10 and it is further reduced by day 30 after first injection in *Smed-AKT(RNAi)* animals. Gene expressions are all relative to the internal control, the ubiquitously expressed clone *H.55.12e*. Graphs represent mean ± s.e.m. of triplicated samples of two or more biological replicates with at least ten animals per experiment. Significance **** <0.0001 is determined through two way-ANOVA. **b** Images show control (top) and *Smed-Akt (RNAi)* (bottom) at day 30 after first dsRNA injection. Images represent depth-coded maximum projections of confocal z-sections in the posterior specific ventral cilia and proximal structures stained with Ac-Tubulin antibody. Superficial structures were labeled in magenta and structures near the dorsal epithelial layer appear in orange (color code scale on the left). Magnified images (on the right) display the effects of *Smed-AKT(RNAi)* on ciliated structures (cilia in ventral epithelia is represented in magenta and sections of the protonephridia in green and blue). Box with dotted lines outline regions of cilia in ventral epithelia, notice the scattered magenta signal in the experimental group. Confocal z-sections were taken on three or more animals of two independent experiments. Depth-coded projections were obtained using ImageJ software. Scale bar 100μm

A distinctive macroscopic feature of the *Smed-Akt (RNAi)* phenotype is the impairment of locomotion accompanied by the elongation of the whole body, which are initiated as early as day 15 and progress for over 35 days after the first dsRNA injection (Additional file 2). Planarian gliding is mediated by synchronized cilia movement on the ventral epithelial surface of the animal [33]. Since locomotion is impaired in *Smed-Akt(RNAi)* animals, we therefore evaluated the status of cilia through the expression levels of genes specifically corresponding to intraflagellar transport machinery and flagellar beating (*IFT88, IC2, LC1, LRRC50* and *DNAHβ-1*) [34, 35]. First, we observed through qPCR, a dramatic reduction in the expression of cilia markers required for the structural and mechanical integrity of cilia [34, 36] (Fig. 4a). The reduction in gene expression of cilia markers is detected as early as 10 days after *Smed-Akt (RNAi)* and their expression continues to reduce over time. Second, whole-mount staining with anti-α-Ac-tubulin antibody allowed us to visualize the integrity of ciliated structures in the ventral epithelia, including parts of the excretory system (e.g. proximal tubules and flame cells in protonephridia) [22, 33–37]. We noted that one month after *Smed-Akt(RNAi),* the anti-α-Ac-tubulin antibody signal is nearly absent in the areas corresponding to ventral cilia, while control animals showed dense coverage by cilia (magenta signal in Fig. 4b). The confocal stacks, 30 days after *Smed-Akt(RNAi),* showed a marked reduction of ventral cilia, making the proximal tubules and flame cells of the excretory system readily evident (Fig. 4b). Together, the results obtained through gene and protein expression demonstrates that *Smed-Akt* is required for the structural integrity of cilia and its maintenance. Additionally, our findings suggest that the impaired locomotion in the Akt phenotype is most likely a consequence of inadequate cilia density in the ventral epithelia.

**Fig. 5:**
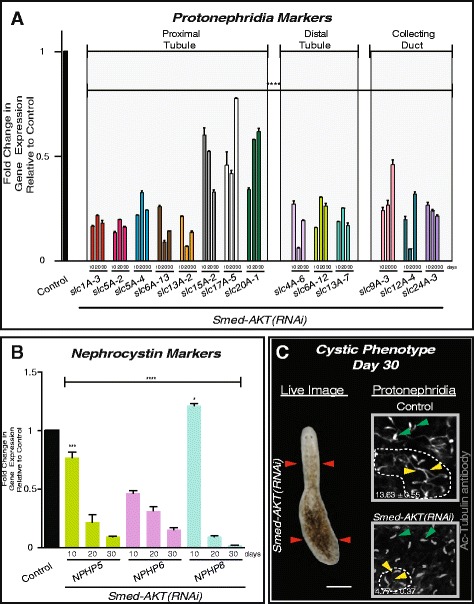
Down-regulation of Akt leads to a delayed onset of a cystic phenotype. **a** Levels of expression of solute carrier (slc) genes expressed along the planarian protonephridia tubules (proximal and distal) and collecting ducts. There is a striking reduction in expression of all slc genes evident as early as day 10 and is sustained through day 30 after *Smed-AKT(RNAi)*. Each individual sample was analyzed relative to its individual control. **b** Expression levels of nephrocystin markers (NPHP5, 6 and 8) at different time points after RNAi. Levels of expression for each gene are relative to individual controls. All NPHP genes showed markedly reduction in gene expression across 30 days after the first dsRNA injection. In all cases, gene expressions are relative to the internal control, the ubiquitously expressed clone *H.55.12e*. Graphs represent mean ± s.e.m. of triplicated samples of two biological replicates consisting of at least ten animals per experiment. Significance **** <0.0001 is determined through two way-ANOVA. **c** Cystic phenotype is seen in *Smed-AKT(RNAi)* animals by day ≥30 after first dsRNA injection (*N* = 30/30). In the live image, seen on the left, the features of a cystic phenotype (elongation of the head and bloating of the tail) are highlighted by red arrows. Confocal z-stack images on the right, control (top) and *Smed-AKT(RNAi)* (bottom), highlight protonephridial clusters (dotted lines)using Ac-Tubulin staining in the posterior ventral surface at day 30. Protonephridial clusters contain tubules (yellow arrows) and flame cells (green arrows). The number of flame cells per proximal unit are strongly reduced (**** *P* < 0.0001, student *t*-test) in *Smed-AKT(RNAi)* animals 4.77 ± 0.72 vs 13.63 ± 0.55 in control. Flame cell counts were obtained from 5 or more animals, totaling N=30 or more clusters analyzed per condition

We next sought to evaluate whether the *Smed-Akt (RNAi)* effects are specific to the ventral epithelia or if they extend to other tissues. The excretory system in planarians consists of protonephridial tubules including both ciliated and non-ciliated structures that could be labeled with anti-α-Ac-tubulin antibody and the *Smed-CAVII-1* gene, respectively [36–38]. Additional markers to each portion of the protonephridial tubules were recently mapped [36], thus providing better opportunities to analyze effects on the excretory system. Specifically, we evaluated the expression of solute carrier transporters (slc) family of genes expressed along the proximal and distal tubules, and the collecting ducts of the protonephridia [36]. We further extended the analysis to include planarian homologs of nephrocystins known to regulate excretory functions and upon their downregulation, lead to cyst-like disease in *S. mediterranea* [36]. The results provided evidence of a generalized reduction in gene expression throughout the protonephridial structures and the nephrocystin markers (NPHP5, 6 and 8) (Fig. 5a, b). The striking reduction in gene expression in the protonephridial structures is evident as early as 10 days after *Akt* disruption. However, macroscopic signs of excretory system defects such as edema and clearing of body pigmentation are evident in *Smed-Akt (RNAi)* animals (30/30) during advanced stages of the phenotype, i.e. ≥ 30 days after the first dsRNA injection (Additional file 3A). Formation of the edema phenotype is consistent with the markedly downregulation of nephrocystin genes, and structural alterations in the proximal structures of the protonephridia (Fig. 5c). Particularly, *Smed-Akt(RNAi)* lead to a generalized decrease in both (13.63 ± 0.55 vs 4.77 ± 0.37) and proximal tubules per protonephridial unit (protonephridial unit is outlined in Fig. 5c), when compared to control. This structural disruption is consistent with the manifestation of edema in planarians [36–38], further validating the reduced integrity of the excretory system during the advanced stages of the *Smed-Akt(RNAi)* phenotype.

**Fig. 6:**
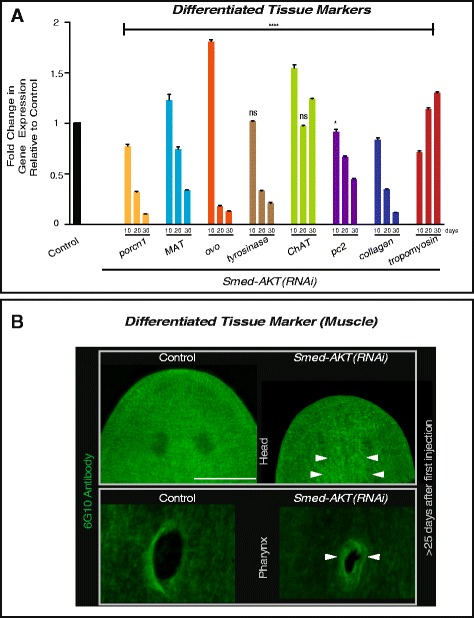
*Smed-AKT(RNAi)* leads to a generalized reduction in the expression of genes in differentiated tissues and alterations in muscle fibers. **a** Gene expression analysis of differentiated tissue markers at days 10, 20 and 30 after first dsRNA injection. All measurements are relative to the their respective control. Analysis of genes related to the differentiated tissues: intestinal (*porcn-1 and MAT*), photoreceptors (*ovo and tyrosinase*), central nervous system (*ChAT and pc2*), and connective and muscle tissues (*collagen and tropomyosin, respectively*). *Smed-AKT(RNAi)* strongly reduce the expression of most markers except for *ChAT* and *tropomyosin* that are elevated by day 30 after RNAi. Gene expressions are all relative to the internal control, the ubiquitously expressed clone *H.55.12e*. Graphs represent mean ± s.e.m. of triplicated samples of two or more independent experiments with at least ten animals per experiment. Significance (*P<0.05 and ****P<0.0001) was determined with two way-ANOVA. **b** Whole-mount immunostaining of intact control and *Smed-AKT(RNAi)* planarians at >day 25 post RNAi initiation with SMED-6G10 (muscle tissue) antibody. SMED-6G10 antibody specifically labels the circular and diagonal muscle fibers throughout the animal. When compared to the control, *Smed-AKT(RNAi)* showed disarray in the muscle fibers in both the head (top images) and pharyngeal (bottom images) muscular structures (arrowheads). The images are representative of an experiment with five animals in one biological replicate. Scale bar 200 μm

To determine whether *Smed-Akt(RNAi)* also affects other organs within planarians, we set out to explore gene and protein expression of markers in terminally differentiated tissues [21, 23, 39–43]. Specifically, mRNA levels of genes associated with the nervous and digestive system, connective and muscle tissues. The results from these experiments demonstrated that most of the genes screened (i.e. 8/10) tend to gradually deplete after *Smed-Akt(RNAi),* but at least two of the markers including the choline acetyltransferase *ChAT* (neurons) and *tropomyosin* (muscle) followed a different pattern characterized by an increase in expression one month after Akt downregulation (Fig. 6a). Staining with the monoclonal antibody Smed-6G10 (6G10) that labels different muscle fibers in the planarian body [44], revealed the disruption of the Akt signaling affects planarian musculature. Specifically, we observed the disorganization of circular and diagonal muscle fibers and the absence of signal in some areas, suggesting alterations in the normal tissue architecture (arrows in head and pharynx, Fig. 6b). The structural changes in musculature may also explain the incapability to ingest food after 20 days of Akt-RNAi (data not shown) but also the animal’s ability to slightly maneuver through peristaltic muscle contractions [34]. ISH experiments also confirmed the widespread reduction in expression of markers of the excretory (*Smed-CAVII-1)*, and the nervous (*Smed-PC2)* system, further supporting the notion that disruption of tissue integrity is not restricted to one tissue in particular but rather a more generalized event after downregulation of Akt function (Additional files 3B, and 4). Future experiments are needed to determine whether particular cell types are more susceptible to structural alterations after Akt systemic inhibition. Nonetheless, these results together demonstrate *Smed-Akt* is essential for the maintenance of tissues in planarians.

### *Smed-Akt(RNAi)* Leads to Regeneration Defects

**Fig. 7:**
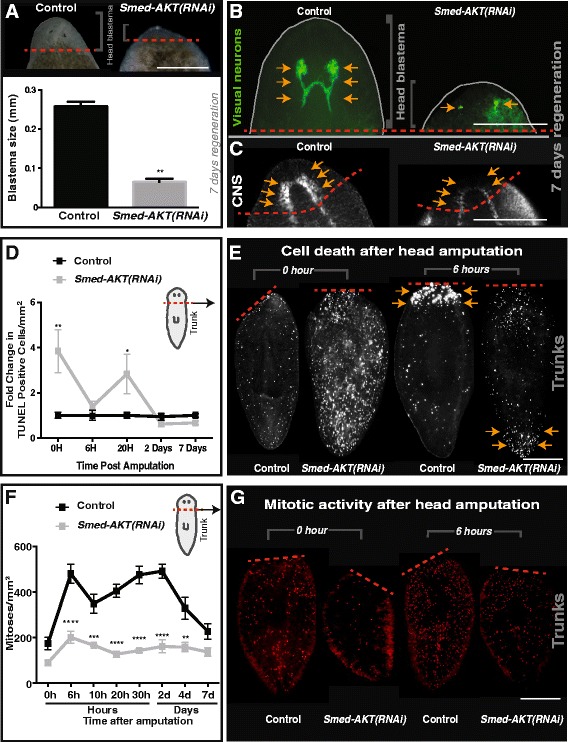
Akt is required for tissue regeneration. **a** Live images of control and *Smed-Akt (RNAi)* head blastema formation seven days post amputation. Dashed red line represents the amputation plane. Scale bar 100μm. **b** Immunostaining labeling the visual neurons (anti-VC-1 antibody) and (**c**) central nervous system (anti-SYNORF1 antibody) seven days post amputation. Arrows highlight these structures in both the control and *Smed-Akt (RNAi)* animals and red dotted lines represent the plane of amputation. Scale bar 100μm. **d** TUNEL-positive nuclei post amputation, represented in fold change compared to the control. Note that cell death decrease over time, a four fold difference in *Smed-Akt (RNAi)*. **e** Immunostaining of TUNEL-positive nuclei of trunk fragments 0 and 6 h post amputation. Scale bar 200 μm. **f** Levels of H3P positive cells over time post amputation in control and *Smed-Akt (RNAi)*. The quantification was performed on the regenerating trunk fragment. **g** Representative images showing mitotic activity (red dots) after head amputation at 0 and 6 h in trunk fragments. All images are representative of two or more biological replicates consisting of five animals or more per experiment. Scale bar 200 μm. Graphs represent mean ± s.e.m. of two or more biological replicates and *P* values *<0.01, **<0.001, ***<0.0005 and ****<0.0001 were obtained with Wilcoxon test or two way ANOVA

Tissue amputation triggers well-coordinated waves of apoptosis and cellular proliferation aimed at recreating missing tissues and organs within the regenerative blastema. The *Smed-Akt* phenotype is characterized by a reduction in neoblast numbers and increased cell death, affecting the maintenance of differentiated tissues. Thus, we assessed how an unbalance in cell death and proliferation affects large-scale injury-induced regeneration in *S. mediterranea*. We performed planarian head decapitations after 30 days post-RNAi initiation and followed macroscopic and microscopic responses in regenerative body trunks (Fig. 7). One week after amputation control animals formed head blastemas with photoreceptor pigmentation, whereas *Smed-Akt(RNAi)* animals only formed a marginal blastema with limited eye pigmentation (Fig. 7a). Further experiments with antibodies that recognize brain structures and visual neurons (i.e. anti-SYNORF1, anti-VC1), revealed *Smed-Akt(RNAi)* animals failed to regenerate brain and visual neuronal connections (Figs. 7b, c). Likewise, animals with tail amputation also fail to regenerate during the advanced phenotype, suggesting that the reduced number of neoblasts may affect both anterior and posterior regeneration in *Smed-Akt(RNAi)* animals (data not shown). These results also imply that injury-mediated cell differentiation is active despite the initial high levels of cell death and low levels of cellular proliferation.

Previous studies in *S. mediterranea* have shown that localized waves of cell death concentrate to the injury site and a systemic spike of neoblast division take place within the first six hours post-amputation [30, 43]. We followed both cell death and the mitotic response post amputation for seven days and observed cell death is suppressed over time, whereas cell division is increased in *Smed-Akt(RNAi)* animals when compared to the initial time point (Figs. 7d-g). Moreover, cell death not only reduced in *Smed-Akt(RNAi)* animals but it also failed to localize to the injury site as is expected in the first six hours post-amputation (Fig. 7d, e and Additional file 4A, B). The system wide cell death expected was also absent even after more than 2 days of amputation (Additional file 5A-C). Instead, TUNEL positive cells were slightly accumulated to the uninjured, opposite end of the regenerating trunk (*n* = 10/10). Intriguingly, amputation in the *Smed-Akt(RNAi)* group elicited a system-wide increase in cell division that peaked at six hours post-amputation and was sustained during the first week post-amputation (Fig. 7f, g). Additionally, the experimental group was incapable of producing a timely and localized mitotic response at the injury site, as seen in the control animals 2 days post amputation. However, *Smed-Akt(RNAi)* animals exhibited a delayed localization of cell division (i.e. 4 days post amputation), which persisted through 7 days post amputation (Additional file 6A-C). We propose *Smed-Akt* functions as regulator of cell death and proliferation, during large-scale tissue regeneration in planarians.

## Discussion

Maintenance of adult tissues proceeds through a fine balance between cell death and cell division. Our results implicate *Smed-Akt* as a critical regulator of cellular turnover, adult tissue maintenance, and regeneration. Disrupting *Smed-Akt* signaling affects the number of proliferative neoblasts during cellular turnover and alters integrity and function of differentiated tissues. Strikingly, tissue injury is capable of altering patterns of cell death and cell proliferation after Akt downregulation.

The *Smed-Akt* phenotype is characterized by an initial increase in cell division that is followed by a gradual depletion in the number of proliferative neoblasts. The nature of the signals triggering cell proliferation in the first 10 days of the phenotype are not clear, but it might involve a compensatory response to overcome deficiencies in cellular turnover due to abnormalities in ciliogenesis [45–48]. Interestingly, even at late stages of the Akt phenotype some neoblasts continue dividing probably to self-renew and/or to continue supporting tissue turnover to some extent as animals subjected to *Smed-Akt(RNAi)* survive for over one month. Two non-exclusive scenarios may explain the presence of dividing neoblasts after *Smed-Akt(RNAi):* (1) residual Akt expression after RNAi, due to incomplete abrogation. Our qPCR analyses demonstrated that *Smed-Akt* expression is strongly downregulated after RNAi, however, additional experiments involving specific antibody against SMED-AKT protein may be needed to rule out whether AKT protein is still present and active, and (2) intrinsic differences among neoblast populations may confer survival properties to a select group of stem cells when Akt is downregulated. This possibility is supported by the differential expression displayed by neoblast subclasses after *Smed-Akt(RNAi)* (Fig. 2b). Recent progress to unravel the diversity of neoblast sub-populations suggest planarian stem cells are more complex than previously anticipated [25, 49]. Therefore, we envision future experiments would evaluate individual neoblast subpopulations to identify whether some neoblast subtypes are more susceptible to Akt downregulation [3, 9, 10, 50, 51].

The increased amount of cell death in *Smed-Akt (RNAi)* suggests this is a rather generalized event involving both neoblasts and differentiated cells. Our findings implicate decisions of cellular survival greatly depend on *Akt* signaling, but future experiments are required to discern whether apoptosis and/or necrosis initially target neoblasts. This possibility may, in fact, reduce the number of neoblasts making them unable to efficiently support demands of cellular turnover, leading to generic tissue defects. We did not address the mechanism of cell death in the *Smed-Akt* phenotype but since the mitochondrial pathway of apoptosis is remarkably conserved in planarians; it may serve, as in vertebrates, as the favored mechanism for *Akt*-mediated cell death [2, 13, 30, 31, 52, 53].

Studies in mammals show that not all tissues respond homogeneously in response to Akt deficiency, while some do not show measurable changes (e.g. bone marrow and pancreas), others undergo apoptosis (e.g. testes and thymus) [52]. A fine balance between stem cell proliferation and programmed cell death enables physiological cellular turnover to support adult tissue maintenance. Tissue renewal is seen to be altered in *Smed-Akt(RNAi)* animals, most likely due to the increased levels of cell death, which reduce the number of proliferating neoblasts. Our findings indicate that *Smed-Akt(RNAi)* lead to a heterogeneous gene expression response and mixed effects on the architecture of differentiated tissues. This differential sensitivity to *Smed-Akt* deficiency is observed early with alteration of cilia-mediated locomotion and the late onset of a cystic phenotype defined by a malfunctioning excretory system. The effects of abrogated *Smed-Akt* in differentiated tissues may depend on tissue-specific turnover rates but more experiments are needed to better understand the phenotype at systemic level.

The mechanisms by which cilia is strongly reduced after *Smed-Akt(RNAi)* is unclear but our findings indicating severe downregulation in IFT88 may suggest, as in other systems [45–47, 54, 55], that phosphorylated Akt fail to localize to the cilium at the cilliary base leading to disruption of the apical cellular projections. An intriguing finding of the *Smed-Akt(RNAi)* phenotype is the disruption of ciliated structures within the epithelium and excretory system (e.g. flame cells and proximal tubules), which is being recently introduced as an alternative model for cystic kidney disease [36]. Our results showed a significant reduction in gene expression correlating to ciliated structures, protonephridia and nephrocystins within the first 10 days of RNAi initiation that persisted to decrease by day 30. Interestingly, impaired locomotion in *Smed-Akt(RNAi)* animals became slightly evident by day 15 after RNAi treatment, which was exacerbated over time, and the delayed onset of the edema phenotype that correlated with a dysfunctional excretory system. Planarian studies have shown functional downregulation of genes correlating to ciliated structures and nephrocystins led to a rapid edema phenotype by 9–15 days post RNAi treatment [36–38]. These results imply that Akt activity may facilitate the assembly of ciliated structures by regulating gene and protein expression required for maintenance of these structures (e.g. IFT88, NPHPs and SCL family of genes). It is also possible that during the initial part of the Akt phenotype, functional protonephridial units balance electrolyte and carry on waste excretion to compensate the reduction in the number of flame cells and proximal tubules. However, as the phenotype progresses it become unsustainable deriving in extensive damage, leading to the collapse of the excretory system. Further experiments will be required to discriminate how Akt signaling regulates the delayed onset of cyst-like formations. Nonetheless, these results are also significant because Akt signaling and cilia are emerging as possible therapeutic target in leukemia and polycystic disease [56–59]. Altogether, these findings highlight the convenience of the planarian model for analyzing Akt signaling dysfunction in the whole adult organism.

Akt has been studied extensively in the context of cancer and as a regulator of cellular functions, but its participation in large-scale regeneration remains poorly understood. Our findings reveal that Akt plays critical roles during planarian regeneration. Specifically, disruption of Akt impairs the process of blastema formation but does not prevent the initial peaks of cell proliferation upon amputation. In response to amputation, some cells migrate and differentiate to form an incipient blastema, which is discontinued probably due to the lack of timely localization of cell death near the wounded area. The mechanisms by which cell death regulates the process of regeneration still remains poorly understood [60]. Nonetheless, the Akt phenotype presents unique opportunities to address whether a particular signaling pathway and/or cell type that plays major role in guiding injury-induced apoptosis. We propose that Akt signaling serves as mediator of localized cell death events during planarian regeneration.

The intriguing finding that injury-induced repair signals in *Smed-Akt(RNAi)* is capable of reducing cell death is exciting and it reveals a novel role for Akt in large-scale tissue regeneration. Uninjured animals subjected to *Smed-Akt(RNAi)* show high levels of cell death and restricted neoblast division, but within a few hours after amputation, levels of apoptosis dramatically reduce while cellular proliferation increase in the absence of functional Akt. While future experiments will be required to investigate the mechanisms contributing to injury-mediated cellular death, these results imply tissue damage and repair may alter cellular decisions imposed by a dysfunctional Akt pathway.

## Conclusions

Our results demonstrate that Akt forms part of an ancient signaling pathway controlling cellular fate decisions in members of the lophotrocozoans. Thus, we introduce *S. mediterranea* as a valuable model system to dissect Akt function in stem cell-based cellular turnover and repair of adult tissues. The mechanisms controlling the molecular cascade guiding large-scale tissue regeneration are poorly understood however, our analysis identified that Akt is necessary for events related with cell death during regeneration. Future experiments are needed to address the injury-mediated signals capable of reverting levels of cell death and proliferation in the absence of Akt signaling.

## Methods

### Planarian Culture

The clonal lines of the Planarian strain, *Schmidtea mediterranea* CIW4 was used for all experimental procedures and were cultured as previously described [61].

### RNAi Experiments

The synthesis of dsRNA was carried out as previously described in [62] and microinjection experiments were carried out following the schedule in Fig. 1a. Following this injection schedule, phenotype was accomplished by 25–30 days post first injection. All experiments were conducted 5 days after last injection.

### Fixation and Whole Mount Immunofluoresence

Animals were fixed using the Carnoys fixation protocol [63] unless otherwise stated. Primary antibody concentrations were used as follows: α -H3P 1:250 (Millipore Cat# 05-817R); α -VC1 1:10,000 (Kind gift of K. Watanabe); anti-α-Ac-Tubulin 1:500 (Sigma, clone 6-11B-1), Smed-6G10 1:1000; SYNORF1 1:100 (Developmental Studies Hybridoma Bank). Secondary antibody concentrations were: Alexa488 (1:400) goat anti-mouse (Invitrogen Cat# 673781), goat-anti-mouse HRP IgG 1:1000 (Life Technologies), and Alexa568 (1:800) goat anti-rabbit (Invitrogen Cat# 11036).

### BrdU Staining

Single staining of BrdU staining was performed as previously described [23, 64].

### Flow Cytometry Analysis and Cell Sorting

Planarians were dissociated as previously described [24, 32]. Brielfy, 1X10^6^ cells from dissociated planaria were stained with DNA marker Draq5 (eBioscience Cat # 65-0880-96) at a 1:500 dilution in CMF media for 30 min at RT in the dark. Incubation with calcein (Invitrogen Cat # C3100MP) 1:500 diluted in CMF media for 10 min at RT was sufficient to stain live cells. BD *FACSDiva*™ software was used for initial gating and samples were either analyzed using LSRII flow cytometer (BD Biosciences) or sorted using ARIAII flow cytometer (BD Biosciences). Apoptotic cells were identified with Annexin V (Pacific Blue) and 7-AAD (PECy5) staining according the manufacturer’s instructions (eBiosence) and additional modifications found in our protocol [32]. Flow cytometry analyses were performed with FlowJo software (version 8). Further details on this protocol can be found in our previous publication [63].

### TUNEL Assay

Cell death was measured, using the TUNEL assay that labels double stranded DNA breaks with fluorescent tags. Further details on this protocol can be found elsewhere [30]. Animals were mounted and fluorescent images were captured and evaluated with Nikon AZ-100 multizoom microscope and NIS Elements software.

### Quantification of Cellular Events, Planarian Measurements and Imaging Processing

Nikon AZ-100 multizoom microscope and NIS Elements (Nikon) AR 3.2 software was used to record animal behavior and digital images of planarian and/or cellular events within the animal. Whole animal measurements were calculated using the number of cellular events (eg. H3P or TUNEL- positive foci) per millimeter square. Area measurement of planarian size decreases was conducted by using 6 or more independent experiment containing 20 or more animals per experiment. Both the control and *Smed-AKT(RNAi)* animals were photographed using the same magnification and area measurements (per mm) were calculated along the 40 day time course. All areas were averaged across experiments. Average areas taken at the site of amputation were confined to a consistent area with a width of 161.05 pixels and a height of 146.939pixels. Fold change representations were determined by dividing experimental/control conditions. Additional details can be found as previously described [63]. Nikon Eclipse Ti confocal microscope and E Z-C1 software were used to obtain Z-stack images using 20X objective. Z-stacks containing 20 sections at 2-1μm intervals were processed using Image J (1.48v). For all images, Adobe Photoshop and Adobe Illustrator were used to adjust color and brightness.

### Quantitative RT-PCR

RNA extraction and quantitative real-time PCR (RT-PCR) reactions were preformed as previously described [63]. RT-PCR from sorted cells was obtained by dissociating >20 animals per condition to extract RNA and prepare cDNA as described before [63]. Equal amount of cells were sorted from both control and experimental conditions, which in some cases required extra animals in experimental groups to achieve the desired target number of cells. In all cases, gene expressions are relative to the ubiquitously expressed clone H.55.12e [24, 63]. Gene expression corresponds to the mean of triplicated samples of at least two independent experiments with pooled RNA extraction of >20 animals each. Fold change represents standardized expression levels of *Smed-Akt(RNAi)*/Control. Each RNAi time point had its own corresponding control RNA extract.

### Whole Mount In Situ Hybridization (WISH)

WISH and fluorescent in situ hybridization were performed on animals fixed in 5% N-acteyl cistein (NAC) solution. Riboprobes were synthesized using T3 and T7 polymerase and digoxigenin-labeled ribonucletide mix with specific PCR templates as previously described [24, 63]. Further details about WISH protocols are found as previously described [65].

### Statistical Analysis

All graphs are expressed as mean ± s.e.m. Statistical analyses were performed with GraphPad Prism software. *P* value less than 0.05 were considered statistically significant.

### Availability of Data and Materials

The datasets supporting the conclusions of this article are included within the article and its Additional files 1, 2, 3, 4, 5 and 6.

### Ethics approval and consent to participate

The study does not involve human data or vertebrate animals.

## Additional files

Additional file 1:
*Smed-Akt(RNAi)* abrogates the expression of markers of neoblast and their postmitotic progeny. Representative images of fluorescent in situ hybridization of *smedwi*-1 (neoblast marker), *Smed-Prog-1* (early neoblast progeny marker) and *Smed-AGAT-1* (late division progeny marker) reveals an important reduction upon Smed-*Akt(RNAi)*. Animals were fixed 30 days post first injection. Experiments were repeated at least twice with ten animals per experiment. Scale bar 200μm. (PDF 433 kb) Additional file 2: The *Smed*-*Akt(RNAi)* phenotype exhibits a progressive inhibition of locomotion. (A-D) Videos of live planarian under (A) 15 day, (B) 20 days, (C) 25 days and (D) 30 days after first dsRNA injection. As time progresses, the phenotype exacerbates. All videos were taken under the same brightfield magnification. (PDF 199 MB) Additional file 3:
*Smed-Akt(RNAi)* leads to down regulation of genes expressed in the excretory system and cyst-like phenotype. (A) Representative live images taken along the time course post *Smed-Akt(RNAi)*. The control is seen on the top and live images of days 15, 25 and 35 post RNAi initiation show the progression of a cyst-like phenotype (30/30) (elongation of the head and bloating of the tail). Notice at 15 days post RNAi treatment, the planarian is thinned and stretched when compared to the control (50/50). (B) Fluorescent in situ hybridization of *Smed-CAVII-1* (excretory system). The signal for *Smed-CAVII-1* is less intense in experimental than in control, indicated with arrows in the posterior part of the animals upon *Smed-Akt (RNAi)* 30 days after first dsRNA injection. Experiments were repeated at least twice with ten animals per experiment. Scale bar 200μm. (C) Fold change in *Smed-CAVII-1* gene expression relative to the control over the course of 10, 20 and 30 days post first dsRNA injection. Gene expressions are all relative to the internal control, the ubiquitously expressed clone H.55.12e. Graphs represent mean ± s.e.m. of triplicated samples of two biological replicates with at least ten animals per experiment. Significance (*** < 0.0005 and **** < 0.0001) was determined with one way-ANOVA. (PDF 544 kb) Additional file 4:
*Smed-Akt(RNAi)* reduces the expression of CNS marker. (Left) Representative images of fluorescent in situ hybridization of *Smed-PC2* (central nervous system) expression depicts a reduction (yellow arrows) upon *Smed-Akt(RNAi)*. Animals were fixed 30 days after first dsRNA injection. Experiments consisted of two biological replicates with ten animals per experiment. Scale bar 200μm. (Middle) Heat map depicting the intensity of signal generated by *Smed-PC2* expression. For intensity images and graph, low levels of expression are seen in purple and high levels of intensity are seen in red. Reduced *Smed-PC2* expression is also indicated via yellow arrows. (Right) The graph on the right represent the distribution of intensities from the pictures in the middle featuring anterior to the posterior region of the animal (control in orange and experimental in gray). The intensity measurement was obtained from the center of the anterior to the center of the posterior (white line in the middle) vertical line by using Image J software. Scale bar 200μm. (PDF 357 kb) Additional file 5:
*Smed-Akt(RNAi)* animals fails to induce local and system-wide cell death response during regeneration. (A) Quantification of the TUNEL-positive nuclei at the site of amputation at various time points in regeneration, the control (black) and the experimental group (grey). (B) Heat map depicting the intensity of signal generated by TUNEL-positive cells 6 h post amputation at the site of head regeneration (red line depicts amputation plane). For intensity images and graph, low levels of expression are seen in purple and high levels of intensity in TUNEL-positive cells are seen in yellow/red. The graphs on the right represent the distribution of these intensities from the anterior to the posterior region of the amputation site (control in orange and experimental in gray). The intensity measurement was obtained from the area covered by the semi-transparent vertical line by using Image J software. Scale bar 100μm. (C) Immunostaining of TUNEL-positive nuclei of trunk fragments for both the control and experimental group at 6 h (localized cell death response), 2 days (system-wide cell death response) and 7 days (blastema formation) post amputation. Arrows indicate cell death dynamics, proper dynamics (control) and improper dynamics (*Smed-Akt(RNAi)*). Yellow brackets denote the formation of the blastema and its relative size. Scale bar 200μm. All images are representative of two or more biological replicates consisting of five animals or more per experiment. Graphs represent mean ± s.e.m. of two or more biological replicates and *P* values * < 0.01, ** < 0.001, *** < 0.0005 and **** < 0.0001 were obtained with two way ANOVA. (PDF 391 kb) Additional file 6: Delayed mitotic response upon amputation in *Smed-Akt(RNAi)* animals. (A) Mitotic activity at the site of amputation at various time points during regeneration, control (black) and *Smed-Akt(RNAi)* in grey. (B) Heat map depicting the intensity of signal generated by H3P-positive cells 4 days post amputation at the site of head regeneration. For intensity images and graph, low levels of expression are seen in purple and high levels of intensity in H3P -positive cells are seen in red. The graphs on the right represent the distribution of these intensities from the anterior to the posterior region of the amputation site (control in orange and experimental in gray). The intensity measurement was obtained from the area covered by the semi-transparent vertical line by using Image J software. Scale bar 100μm. (C) Whole-mount immunostaining of H3P-positive cells on trunk fragments for both the control and experimental group from 0 to 6 h (system-wide mitotic response), 2 to 4 days (localized mitotic response) and 7 days (blastema formation) post amputation. Arrows indicate cell proliferation dynamics during regeneration, proper dynamics (control) and improper dynamics (*Smed-Akt(RNAi)*. Yellow brackets denote the formation of the blastema and its size. Notice, at 4 and 7 days, mitotic activity is localized to the site of amputation. Scale bar 200μm. All images are representative of two or more biological replicates consisting of five animals or more per experiment. Graphs represent mean ± s.e.m. of two or more biological replicates and *P* values * < 0.01, ** < 0.001, *** < 0.0005 and **** < 0.0001 were obtained with two way ANOVA. (PDF 282 kb)

## Abbreviations

RNAi: RNA-interference
dsRNA: Double-stranded RNA
PI3K: Phosphoinositide 3-kinase
*Smed-Akt*: The planarian Akt ortholog
PTEN: Phosphatase and Tensin homolog
BrdU: Bromodeoxyuridine analog
CDK1-4: Cyclin-Dependent Kinase 1–4
*Prog-1*: Early division progeny −1
*Agat-1*: L-arginine:glycine amidinotransferase marker of the neoblast late division progeny
FACS: Fluorescence-activated cell sorting
anti-α-Ac-tubulin: antibody against α-acetylated tubulin
TUNEL: Terminal deoxynucleotidyl transferase dUTP nick end labeling
*ChAT*: Choline acetyltransferase
SYNORF1: Immunogen sequence against synapsin-1
VC1: Visual cells −1
PC2: Prohormone convertase-2
qPCR: Quantitative polymerase chain reaction
H3P: Phospho-Histone H3 (Ser10) Antibody
WISH: Whole-mount in situ hybridization
s.e.m.: Standard error of the mean
