# Supplementary figures and images for: The Akt signaling pathway is required for tissue maintenance and regeneration in planarians
Source: BMC Dev Biol. 2016 Apr 11;16:7. doi: 10.1186/s12861-016-0107-z (PMC4827215; doi:10.1186/s12861-016-0107-z)

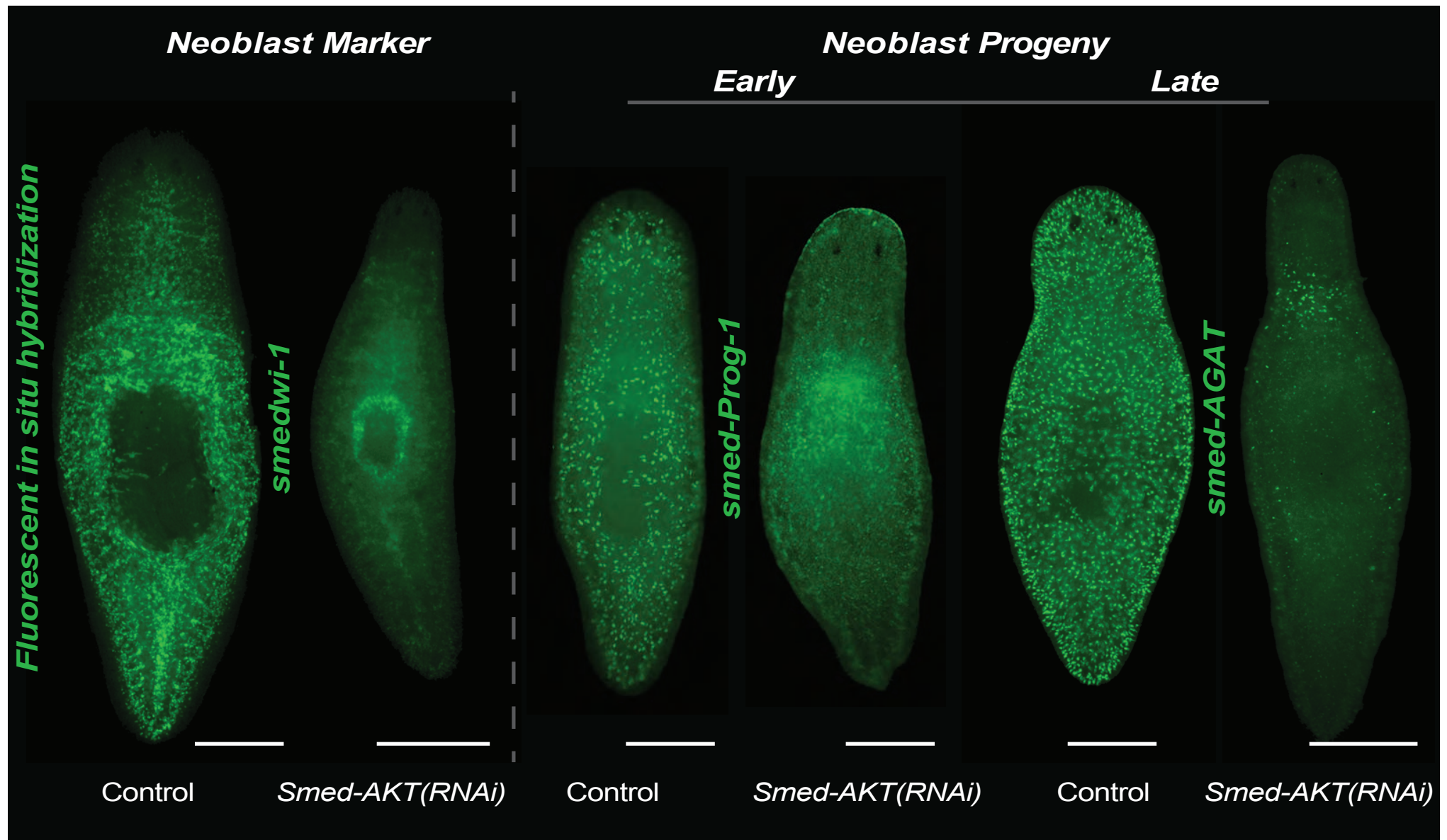

Supplement: Additional file 1: — Smed-Akt(RNAi) abrogates the expression of markers of neoblast and their postmitotic progeny. Representative images of fluorescent in situ hybridization of smedwi-1 (neoblast marker), Smed-Prog-1 (early neoblast progeny marker) and Smed-AGAT-1 (late division progeny marker) reveals an important reduction upon Smed-Akt(RNAi). Animals were fixed 30 days post first injection. Experiments were repeated at least twice with ten animals per experiment. Scale bar 200μm. (PDF 433 kb) [file 12861_2016_107_MOESM1_ESM.pdf]

## *Locomotion defects in Smed-AKT(RNAi)*

*Smed-AKT(RNAi)*

*15 days*

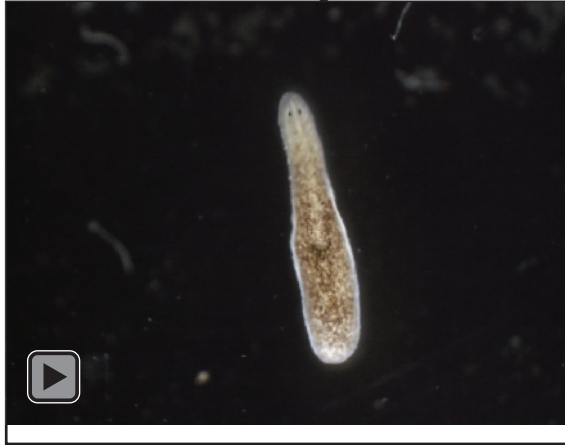

*Smed-AKT(RNAi)*

*20 days*

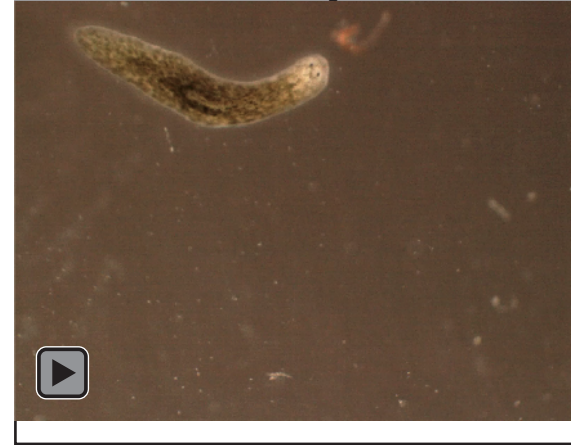

*Smed-AKT(RNAi)*

*25 days*

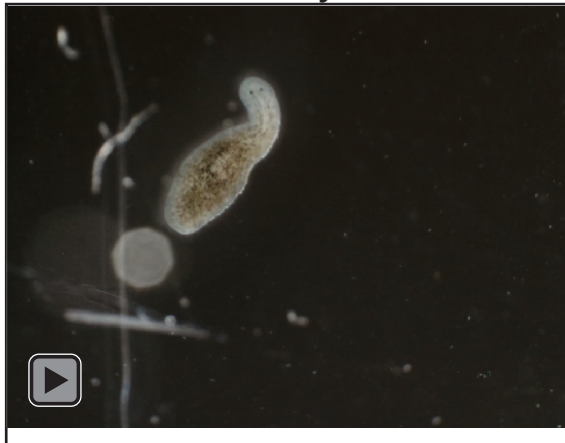

Supplement: Additional file 2: — The Smed-Akt(RNAi) phenotype exhibits a progressive inhibition of locomotion. (A-D) Videos of live planarian under (A) 15 day, (B) 20 days, (C) 25 days and (D) 30 days after first dsRNA injection. As time progresses, the phenotype exacerbates. All videos were taken under the same brightfield magnification. (PDF 199 MB) [file 12861_2016_107_MOESM2_ESM.pdf]

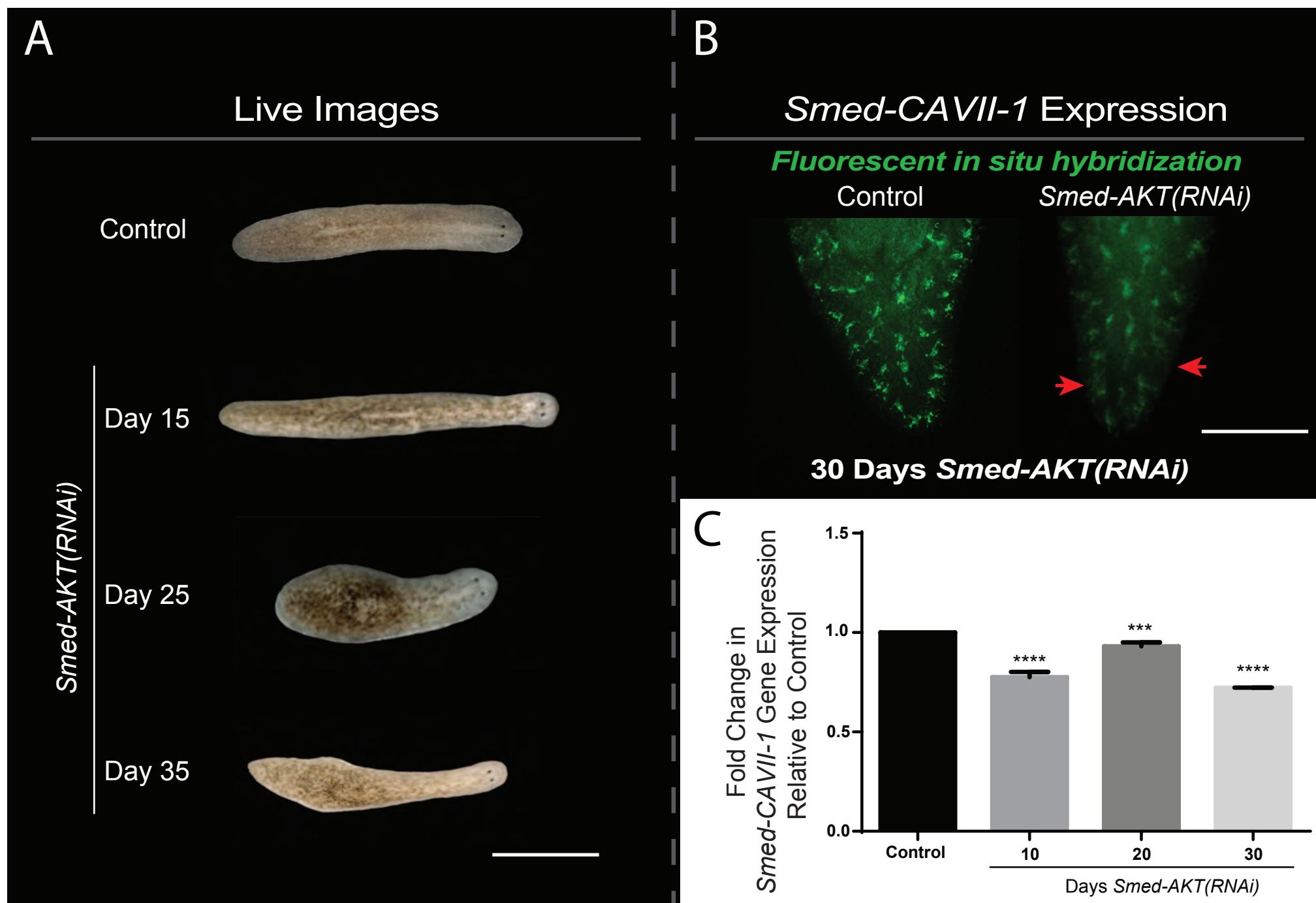

Supplement: Additional file 3: — Smed-Akt(RNAi) leads to down regulation of genes expressed in the excretory system and cyst-like phenotype. (A) Representative live images taken along the time course post Smed-Akt(RNAi). The control is seen on the top and live images of days 15, 25 and 35 post RNAi initiation show the progression of a cyst-like phenotype (30/30) (elongation of the head and bloating of the tail). Notice at 15 days post RNAi treatment, the planarian is thinned and stretched when compared to the control (50/50). (B) Fluorescent in situ hybridization of Smed-CAVII-1 (excretory system). The signal for Smed-CAVII-1 is less intense in experimental than in control, indicated with arrows in the posterior part of the animals upon Smed-Akt (RNAi) 30 days after first dsRNA injection. Experiments were repeated at least twice with ten animals per experiment. Scale bar 200μm. (C) Fold change in Smed-CAVII-1 gene expression relative to the control over the course of 10, 20 and 30 days post first dsRNA injection. Gene expressions are all relative to the internal control, the ubiquitously expressed clone H.55.12e. Graphs represent mean ± s.e.m. of triplicated samples of two biological replicates with at least ten animals per experiment. Significance (*** < 0.0005 and **** < 0.0001) was determined with one way-ANOVA. (PDF 544 kb) [file 12861_2016_107_MOESM3_ESM.pdf]

**A**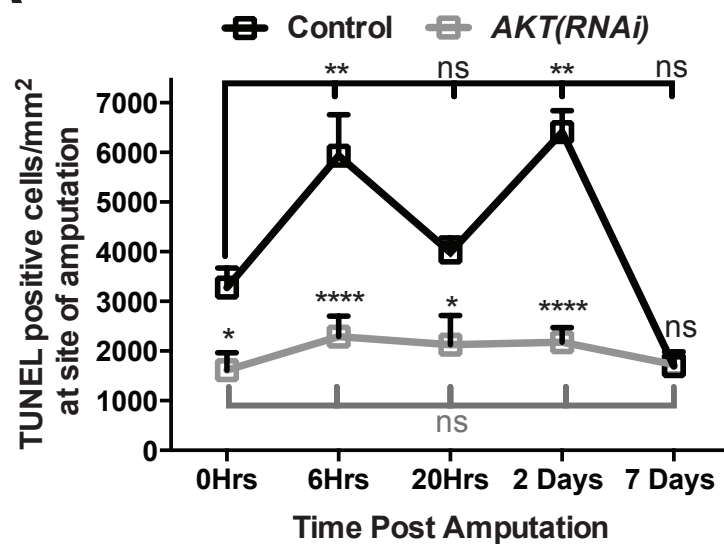**B**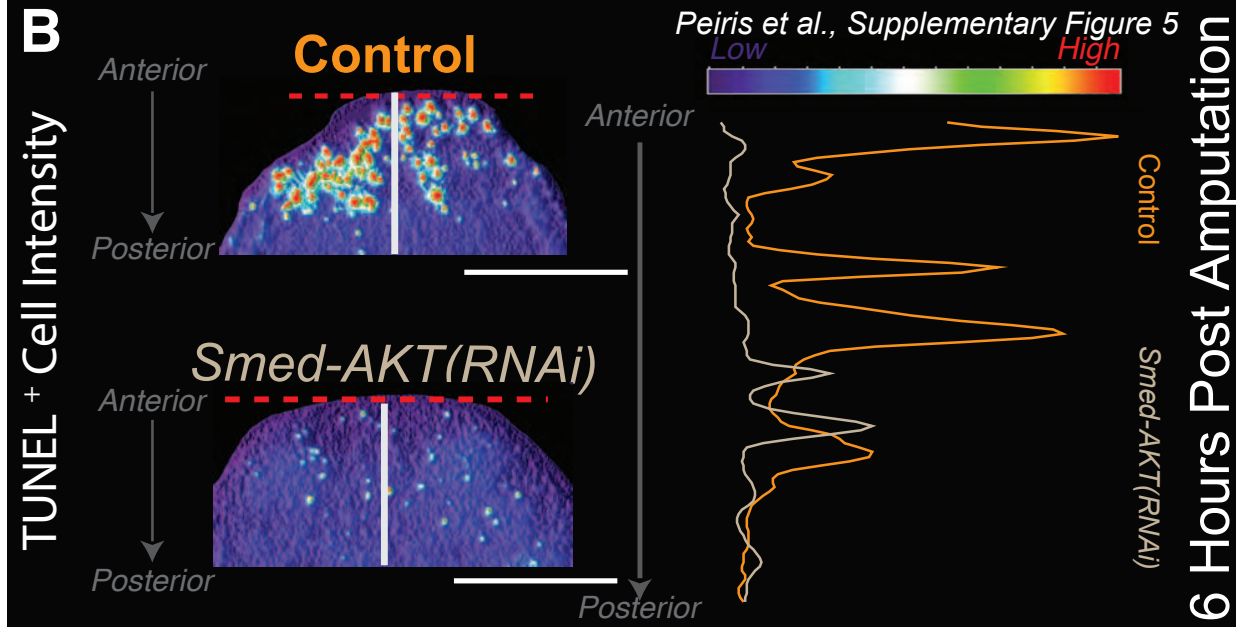**C**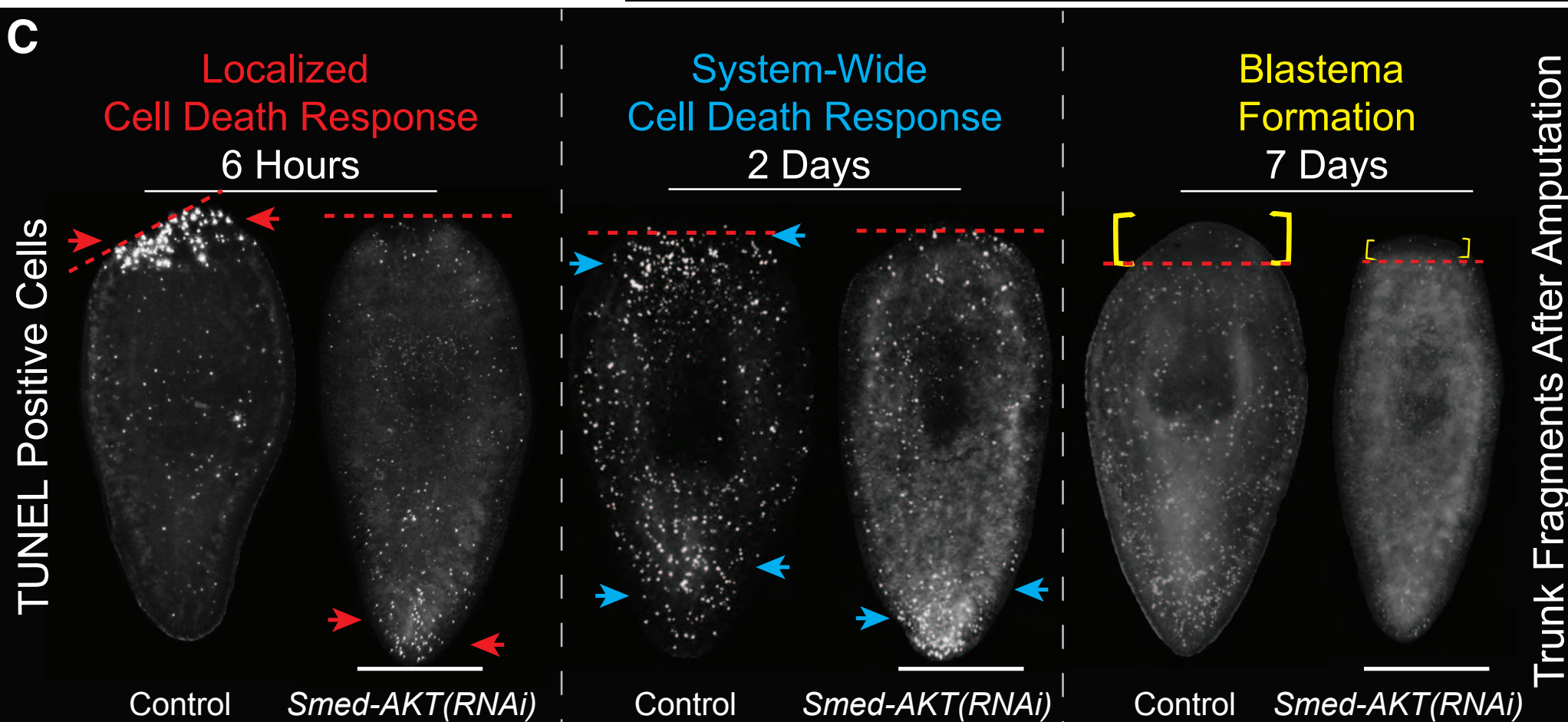

Supplement: Additional file 5: — Smed-Akt(RNAi) animals fails to induce local and system-wide cell death response during regeneration. (A) Quantification of the TUNEL-positive nuclei at the site of amputation at various time points in regeneration, the control (black) and the experimental group (grey). (B) Heat map depicting the intensity of signal generated by TUNEL-positive cells 6 h post amputation at the site of head regeneration (red line depicts amputation plane). For intensity images and graph, low levels of expression are seen in purple and high levels of intensity in TUNEL-positive cells are seen in yellow/red. The graphs on the right represent the distribution of these intensities from the anterior to the posterior region of the amputation site (control in orange and experimental in gray). The intensity measurement was obtained from the area covered by the semi-transparent vertical line by using Image J software. Scale bar 100μm. (C) Immunostaining of TUNEL-positive nuclei of trunk fragments for both the control and experimental group at 6 h (localized cell death response), 2 days (system-wide cell death response) and 7 days (blastema formation) post amputation. Arrows indicate cell death dynamics, proper dynamics (control) and improper dynamics (Smed-Akt(RNAi)). Yellow brackets denote the formation of the blastema and its relative size. Scale bar 200μm. All images are representative of two or more biological replicates consisting of five animals or more per experiment. Graphs represent mean ± s.e.m. of two or more biological replicates and P values * < 0.01, ** < 0.001, *** < 0.0005 and **** < 0.0001 were obtained with two way ANOVA. (PDF 391 kb) [file 12861_2016_107_MOESM5_ESM.pdf]
