# Supplementary material for: The Akt signaling pathway is required for tissue maintenance and regeneration in planarians
Source: BMC Dev Biol. 2016 Apr 11;16:7. doi: 10.1186/s12861-016-0107-z (PMC4827215; doi:10.1186/s12861-016-0107-z)
Supplement: Additional file 4: — Smed-Akt(RNAi) reduces the expression of CNS marker. (Left) Representative images of fluorescent in situ hybridization of Smed-PC2 (central nervous system) expression depicts a reduction (yellow arrows) upon Smed-Akt(RNAi). Animals were fixed 30 days after first dsRNA injection. Experiments consisted of two biological replicates with ten animals per experiment. Scale bar 200μm. (Middle) Heat map depicting the intensity of signal generated by Smed-PC2 expression. For intensity images and graph, low levels of expression are seen in purple and high levels of intensity are seen in red. Reduced Smed-PC2 expression is also indicated via yellow arrows. (Right) The graph on the right represent the distribution of intensities from the pictures in the middle featuring anterior to the posterior region of the animal (control in orange and experimental in gray). The intensity measurement was obtained from the center of the anterior to the center of the posterior (white line in the middle) vertical line by using Image J software. Scale bar 200μm. (PDF 357 kb) [file 12861_2016_107_MOESM4_ESM.pdf]

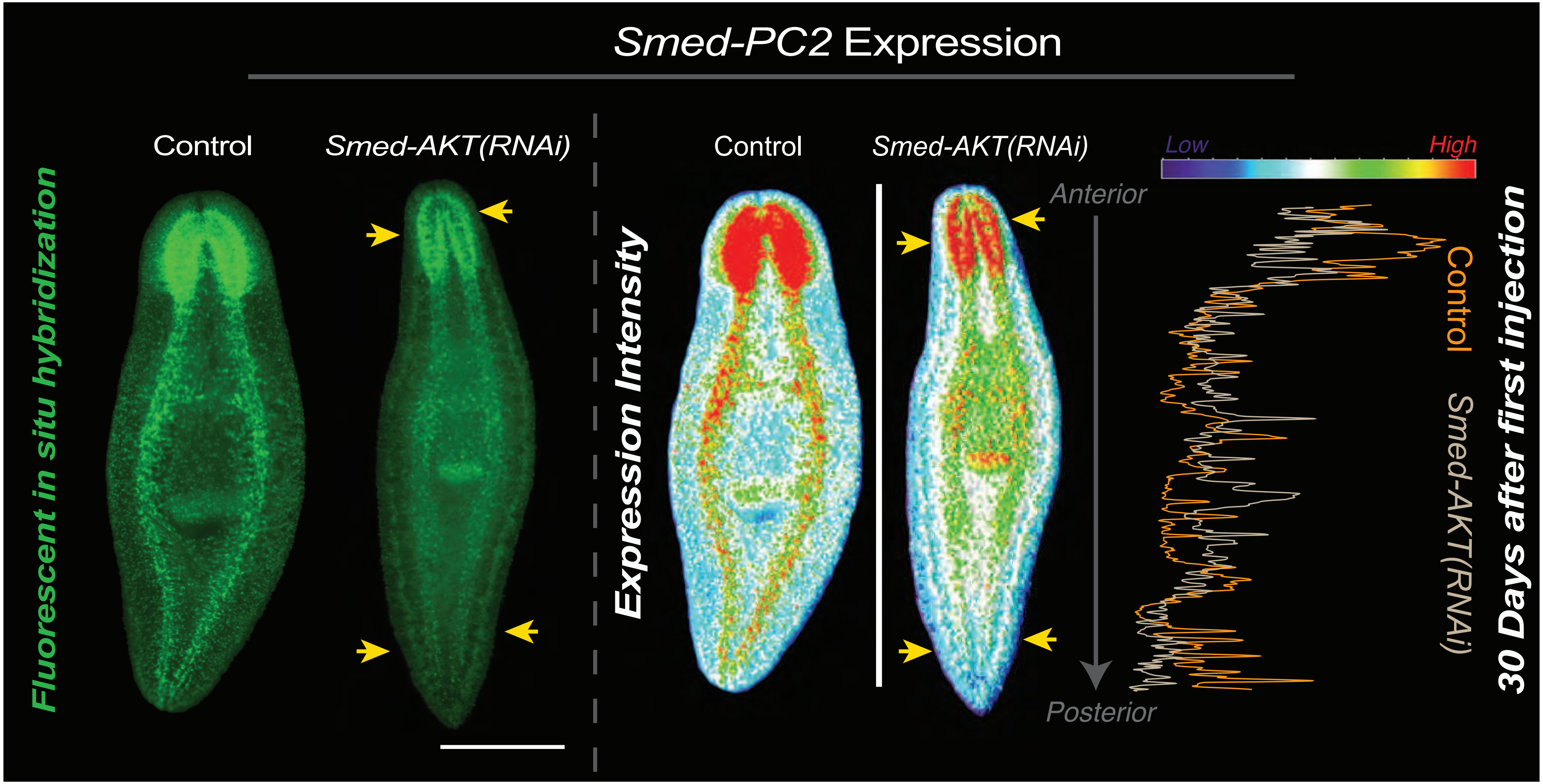

Smed-PC2 Expression
Fluorescent *in situ* hybridization
Control
Smed-AKT(RNAi)
Expression Intensity
Control
Smed-AKT(RNAi)
Low
High
Anterior
Posterior
Control
Smed-AKT(RNAi)
30 Days after first injection
