# Supplementary material for: The Akt signaling pathway is required for tissue maintenance and regeneration in planarians
Source: BMC Dev Biol. 2016 Apr 11;16:7. doi: 10.1186/s12861-016-0107-z (PMC4827215; doi:10.1186/s12861-016-0107-z)
Supplement: Additional file 6: — Delayed mitotic response upon amputation in Smed-Akt(RNAi) animals. (A) Mitotic activity at the site of amputation at various time points during regeneration, control (black) and Smed-Akt(RNAi) in grey. (B) Heat map depicting the intensity of signal generated by H3P-positive cells 4 days post amputation at the site of head regeneration. For intensity images and graph, low levels of expression are seen in purple and high levels of intensity in H3P -positive cells are seen in red. The graphs on the right represent the distribution of these intensities from the anterior to the posterior region of the amputation site (control in orange and experimental in gray). The intensity measurement was obtained from the area covered by the semi-transparent vertical line by using Image J software. Scale bar 100μm. (C) Whole-mount immunostaining of H3P-positive cells on trunk fragments for both the control and experimental group from 0 to 6 h (system-wide mitotic response), 2 to 4 days (localized mitotic response) and 7 days (blastema formation) post amputation. Arrows indicate cell proliferation dynamics during regeneration, proper dynamics (control) and improper dynamics (Smed-Akt(RNAi). Yellow brackets denote the formation of the blastema and its size. Notice, at 4 and 7 days, mitotic activity is localized to the site of amputation. Scale bar 200μm. All images are representative of two or more biological replicates consisting of five animals or more per experiment. Graphs represent mean ± s.e.m. of two or more biological replicates and P values * < 0.01, ** < 0.001, *** < 0.0005 and **** < 0.0001 were obtained with two way ANOVA. (PDF 282 kb) [file 12861_2016_107_MOESM6_ESM.pdf]

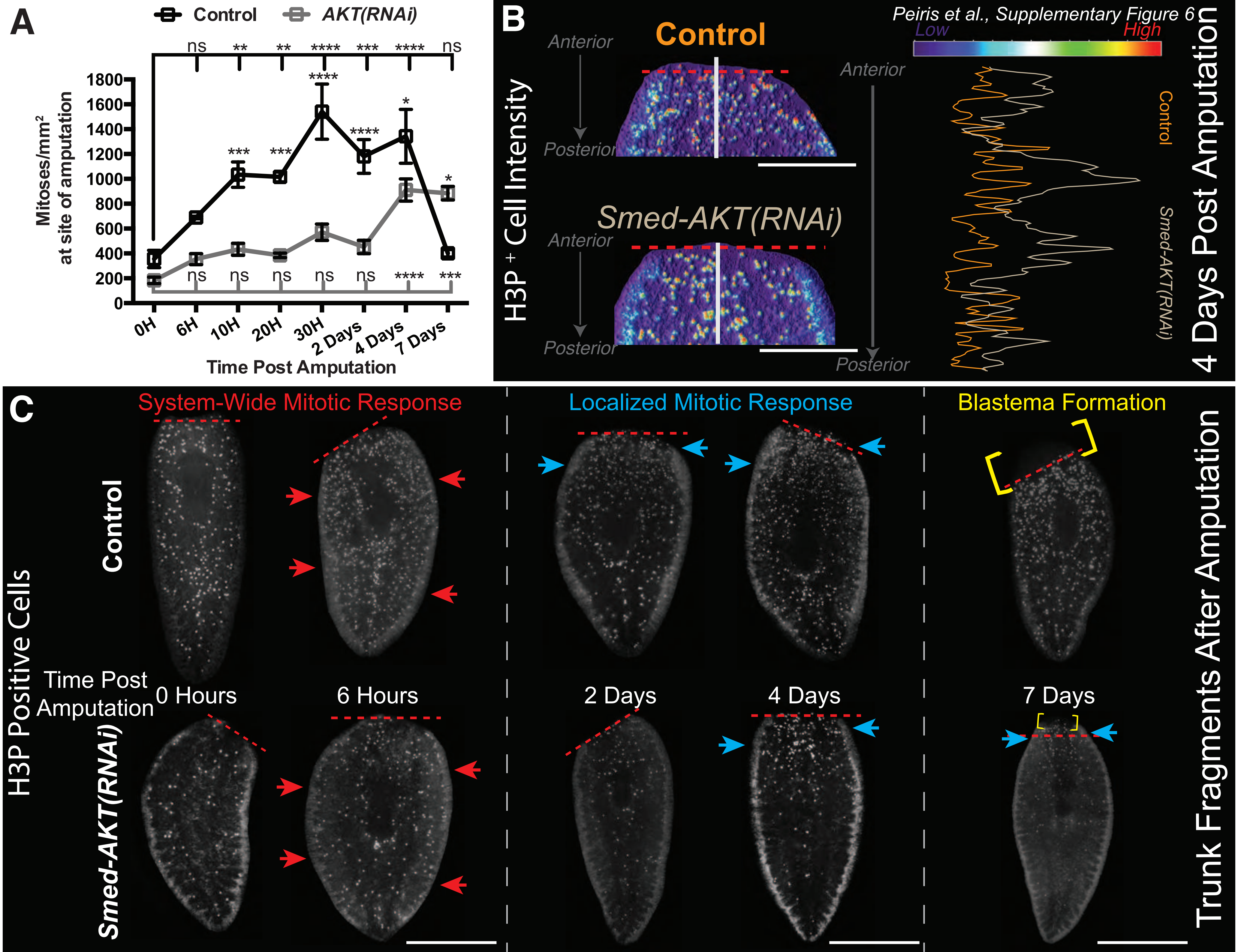

A
Control
AKT(RNAi)
Mitoses/mm²
at site of amputation
Time Post Amputation
0H
6H
10H
20H
30H
2 Days
4 Days
7 Days
B
Peiris et al., Supplementary Figure 6
H3P + Cell Intensity
Control
Smed-AKT(RNAi)
Anterior
Posterior
Low
High
4 Days Post Amputation
C
System-Wide Mitotic Response
Localized Mitotic Response
Blastema Formation
H3P Positive Cells
Time Post Amputation
0 Hours
6 Hours
2 Days
4 Days
7 Days
Trunk Fragments After Amputation
